# Association between frailty trajectories and stroke incidence among Chinese older adults: evidence from CHARLS 2011–2015

**DOI:** 10.3389/fneur.2025.1592565

**Published:** 2025-07-14

**Authors:** Yuanying Song, Jianping Liu, Sufang Wang, Haicun Shi, Lijian Han

**Affiliations:** ^1^Department of Neurology, Yancheng Third People's Hospital (Affiliated Hospital 6 of Nantong University, The Yancheng School of Clinical Medicine of Nanjing Medical University, The Affiliated Hospital of Jiangsu Vocational College of Medicine), Yancheng, Jiangsu, China; ^2^School of Pharmacy, Nanjing Medical University, Nanjing, Jiangsu, China

**Keywords:** frailty, stroke, epidemiology, aging, incidence, prevention

## Abstract

**Background:**

While frailty is recognized as a significant predictor of adverse health outcomes in older adults, the relationship between different frailty assessment approaches and stroke risk remains understudied in the Chinese elderly population.

**Methods:**

Data were derived from the China Health and Retirement Longitudinal Study (CHARLS) 2011–2015, including 8,049 participants. Frailty was assessed using two approaches: cluster analysis (K-means method) based on longitudinal frailty index trajectories and baseline frailty index. Cox proportional hazards models were employed to examine the association between frailty and stroke incidence, adjusting for sociodemographic characteristics and cardiovascular risk factors including age, marital status, education, smoking, drinking, BMI, glycated hemoglobin, systolic blood pressure, HDL cholesterol, C-reactive protein, and physical dysfunction.

**Results:**

During the 5-year follow-up period, 768 stroke events were recorded. In the fully adjusted models, both frailty assessment approaches showed significant associations with stroke risk. The cluster-based approach demonstrated a higher risk of stroke (HR = 3.85, 95% CI: 3.26–4.56, *p* < 0.001) compared to the baseline frailty index (HR = 1.14, 95% CI: 1.12–1.16, *p* < 0.001). Traditional cardiovascular risk factors remained significant predictors in both models. Subgroup analyses consistently demonstrated significant associations across nearly all demographic and health-related subgroups.

**Conclusion:**

Both longitudinal frailty trajectories and baseline frailty index are independently associated with stroke risk in Chinese older adults, with the cluster-based approach showing stronger predictive value. These findings suggest the importance of considering frailty patterns in stroke risk assessment and highlight the potential value of longitudinal frailty monitoring in stroke prevention strategies for aging populations.

## Introduction

Frailty, a condition characterized by the progressive decline in physiological reserves and the increased vulnerability to adverse health outcomes, has become a critical area of research in aging populations (1–3). As populations around the world, including China, continue to age rapidly, frailty and its associated risks, such as stroke, have emerged as significant public health concerns (4–7). Stroke, a leading cause of death and disability globally, is particularly prevalent in older adults, making the identification of factors that contribute to stroke risk paramount (8, 9). Among the various determinants of stroke, frailty stands out due to its multifaceted nature and its potential role in exacerbating cardiovascular diseases, including stroke. Despite the growing recognition of frailty as a key predictor of adverse health outcomes, its relationship with stroke incidence, especially in the context of Chinese older adults, remains underexplored.

China, with its vast aging population, faces an urgent need to address the health challenges posed by aging (10, 11). According to recent estimates, the number of individuals aged 60 years and older in China exceeds 250 million, accounting for nearly 18% of the total population. This demographic shift has been accompanied by a rise in chronic diseases, including stroke, which is now one of the leading causes of death and long-term disability in the country (12). Stroke risk in older adults is influenced by a range of factors, including traditional cardiovascular risk factors such as hypertension, smoking, and dyslipidemia, as well as emerging risk factors like frailty (13, 14). However, the interplay between frailty and stroke risk, particularly how different approaches to frailty assessment influence stroke incidence, remains a critical but understudied area in the Chinese context.

Our study adopts two complementary approaches to assess frailty in older adults: (1) a cluster-based frailty trajectory method that identifies longitudinal patterns of frailty progression across multiple time points, and (2) a baseline frailty index approach that captures frailty status at a single time point. This dual-method framework allows us to compare dynamic versus static assessments of frailty and evaluate their respective contributions to stroke risk prediction. By distinguishing between these assessment strategies, the study provides a more comprehensive understanding of how the temporal dimension of frailty influences stroke incidence in the aging Chinese population.

The China Health and Retirement Longitudinal Study (CHARLS), a large-scale, nationally representative study of adults aged 45 and older, offers a unique opportunity to examine the relationship between frailty and stroke risk in the Chinese elderly population (15). With data spanning from 2011 to 2018, the CHARLS dataset provides valuable insights into the health trajectories of older Chinese adults, including information on frailty, cardiovascular risk factors, and stroke incidence. This study aims to investigate the association between frailty trajectories, assessed using two different methods, and the incidence of stroke among older Chinese adults. Specifically, we will compare the predictive value of a cluster-based approach to frailty, which identifies distinct frailty trajectories over time, with the traditional baseline frailty index, which captures an individual’s frailty status at a single point in time.

## Methods

### Study population

The China Health and Retirement Longitudinal Study (CHARLS) represents a comprehensive, nationally representative longitudinal survey across China. Data collection was conducted through face-to-face interviews utilizing a multistage, stratified probability-proportional-to-size sampling strategy, ensuring appropriate population representation (16). Study participants completed standardized questionnaires that encompassed detailed information on sociodemographic characteristics, lifestyle behaviors, and health-related parameters. The study protocol and methodology are documented in detail on the CHARLS official website. All participants provided written informed consent before enrollment. The study received approval from the Institutional Review Board of Peking University and was conducted in accordance with the ethical principles established in the Declaration of Helsinki. The analytical sample comprised 8,049 participants who were followed from 2011 to 2015.

### Assessment of frailty

Frailty assessment was conducted using the frailty index methodology, which quantifies the cumulative burden of age-associated health deficits. The index was constructed following standardized procedures established in the literature (17). The frailty index was constructed using 32 health-related variables encompassing chronic conditions, sensory impairments, functional limitations (ADL/IADL), self-rated health, depression, and cognitive function. The composite frailty index was constructed by summing the number of present health deficits across 32 items, resulting in a total score ranging from 0 to 32. For missing values, participants were included in the calculation only if they had no more than 3 missing items among the components. For each participant, the frailty index was computed by summing all non-missing deficits and dividing by the number of non-missing items, then scaled to a 0–32 range. If more than three items were missing, the FI was coded as missing and excluded from analysis. In this study, the frailty index was treated as a continuous variable in all analyses, rather than dichotomized into “frail” and “non-frail” categories.

### Assessment of stroke

Stroke incidents were identified through participant questionnaires based on physician diagnoses or documented treatment for stroke during the follow-up period (2011–2015). Participants were followed until either stroke diagnosis, loss to follow-up, or the end of the study period. This standardized approach to stroke ascertainment ensured consistent case identification throughout the study duration. Although stroke ascertainment relied on self-reported physician diagnoses, prior validation studies suggest reasonable accuracy in similar large-scale surveys (18).

### Covariates

The covariates included age, sex, marital status, education, smoking status, drinking status, physical dysfunction, body mass index (BMI), glycated haemoglobin (HbA1c), systolic blood pressure (SBP), high-density lipoprotein cholesterol (HDL-C), and C-reactive protein. Marital status was divided into two categories: married or non-married. Education was classified into three levels: primary school or below, high school, and college or above. Smoking status was categorized as never smokers and ever smokers, drinking status was categorized as never drinkers and ever drinkers. Physical dysfunction was divided into two categories: physical dysfunction or non-physical dysfunction. The selection of these covariates was also based on established associations with frailty or as previously reported in the literature (19, 20).

### K-means clustering

The trajectory patterns of frailty were analyzed using K-means clustering based on three time points of frailty index measurements (2011, 2013, and 2015) from CHARLS. Prior to clustering, the frailty index values were standardized to ensure equal weighting of measurements across time points. The optimal number of clusters was determined using both the silhouette method and elbow method. The silhouette method evaluates clustering validity based on the sample’s proximity to neighboring clusters, while the elbow method examines the percentage of variance explained as a function of the number of clusters. Based on these analyses, a two-cluster solution was selected as the optimal choice. The robustness of the clustering solution was visualized through both trajectory plots and cluster visualization plots. The trajectory plot displayed the temporal patterns of frailty index values for each cluster from 2011 to 2015, while the cluster visualization illustrated the distribution of observations in the multidimensional space using convex hulls to delineate cluster boundaries. The resulting clusters were incorporated into subsequent survival analyses as predictors of stroke risk. And we conducted subsampling-based cluster stability analysis to check the stability of clustering. The analysis was repeated 100 times with 80% subsample fractions using K-means. The mean cluster stability probabilities (bootmean) were used to evaluated the robustness of the clustering solution.

### Statistical analysis

Cox proportional hazards models were employed to examine the association between frailty (both cluster-based and baseline index) and stroke incidence. Four models were constructed: Model 1 examined the unadjusted association between frailty cluster and stroke; Model 2 added sociodemographic and health-related covariates to Model 1; Model 3 assessed the unadjusted association between baseline frailty index and stroke; and Model 4 incorporated the same covariates as Model 2 with baseline frailty index. Covariates included age, gender, marital status, education level, smoking status, alcohol consumption, BMI, glycated hemoglobin, systolic blood pressure, HDL cholesterol, C-reactive protein, and physical dysfunction.

To explore potential effect modifications, stratified analyses were conducted across various subgroups including age (<80 vs. ≥80 years), gender (male vs. female), marital status (married vs. non-married), smoking status (yes vs. no), alcohol consumption (yes vs. no), and physical dysfunction status (yes vs. no). Each stratified analysis employed the same four-model framework as the primary analysis, with appropriate adjustments for potential confounders. All analyses were performed using R version 4.2.3 (R Foundation for Statistical Computing, Vienna, Austria), with the ‘survival’ package for Cox regression analyses. Statistical significance was set at *p* < 0.05 (two-sided).

## Results

### K-means clustering

**Figure 1:**
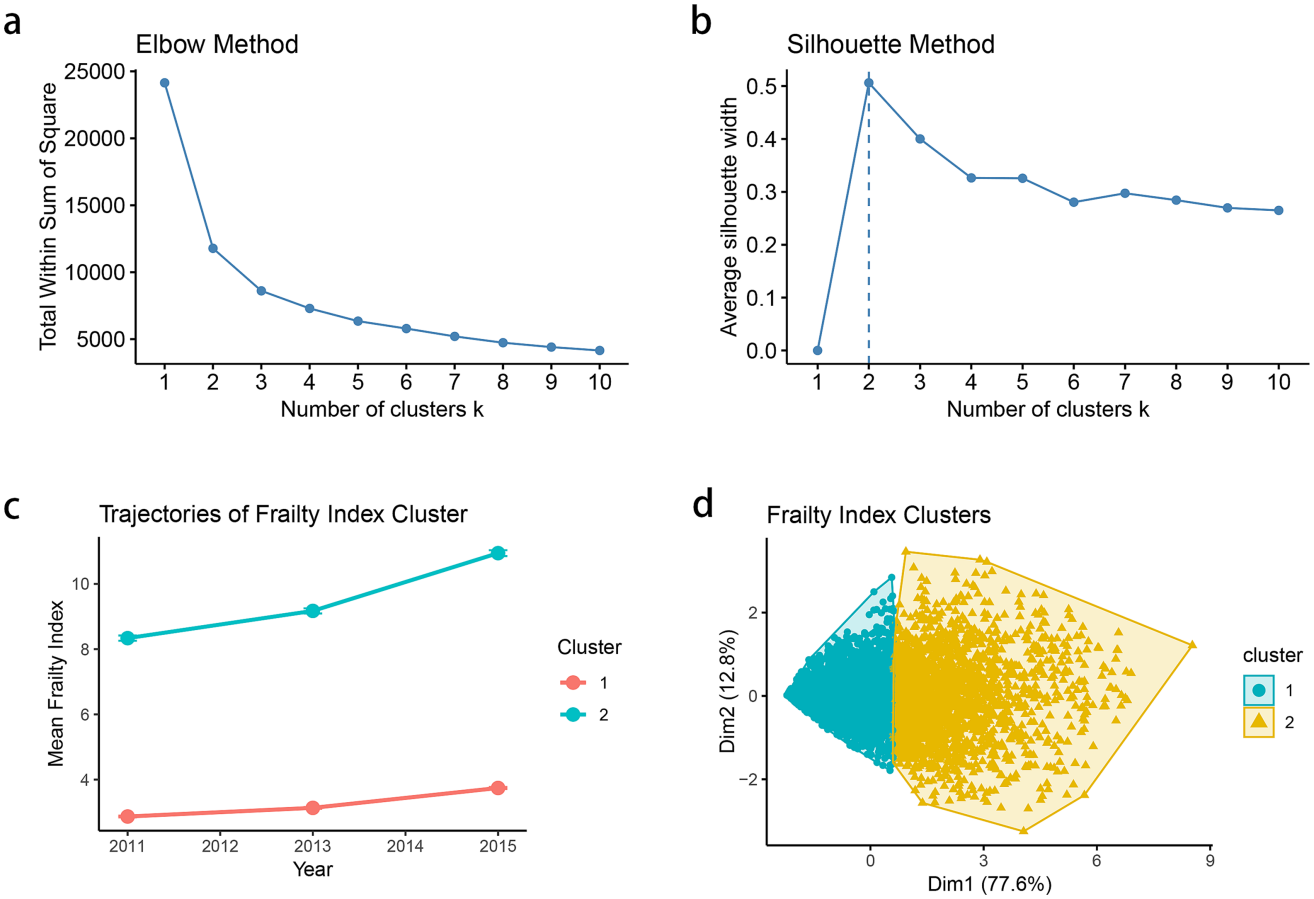
**(a)** Elbow method to determine the optimal number of clusters based on within-cluster sum of squares. **(b)** Silhouette method for cluster validation and average silhouette width is plotted for *k* = 2–10. **(c)** Mean frailty index trajectories from 2011 to 2015 for each identified cluster. Each point represents the mean frailty index for the corresponding cluster at a given time point (2011, 2013, and 2015), and the vertical error bars denote the standard error (SE) of the mean. **(d)** Visualization of cluster separation in two-dimensional principal component space.

The optimal number of clusters was determined using both the elbow method and silhouette analysis. The elbow method showed a substantial decrease in total within-sum-of-squares up to *k* = 2, after which the decrease became more gradual, suggesting a two-cluster solution (Figure 1a). This was further supported by the silhouette analysis, which demonstrated the highest average silhouette width (approximately 0.5) at *k* = 2, indicating optimal cluster separation (Figure 1b). The longitudinal trajectory analysis revealed two distinct patterns of frailty progression from 2011 to 2015. Cluster 1 (represented in red) showed a relatively stable or slightly declining trajectory with lower frailty index values, while Cluster 2 (represented in blue) exhibited an increasing trend with consistently higher frailty index values (Figure 1c). The two-dimensional visualization of the clusters, accounting for 90.4% of the total variance (Dim1: 77.6%, Dim2: 12.8%), demonstrated clear separation between the clusters, with minimal overlap in the feature space (Figure 1d). To evaluate clustering robustness, we applied the clusterboot procedure with 100 iterations and 80% subsampling. The average cluster stability (bootmean) was 0.91 for Cluster 1 and 0.88 for Cluster 2, indicating high robustness of the two-cluster solution.

Beyond differences in frailty index scores, the two clusters exhibited distinct clinical and sociodemographic profiles at baseline (Supplementary Table S1). Compared to Cluster 1, participants in Cluster 2 were more likely to be older (mean age 61.9 vs. 57.1 years), female (65.4% vs. 50.6%), and non-married (16.6% vs. 9.2%), and had significantly lower educational attainment (83.9% vs. 65.8% with primary school or below, *p* < 0.001). Cluster 2 also had a higher prevalence of physical dysfunction (95.7% vs. 58.4%), elevated systolic blood pressure (mean 133.6 vs. 127.2 mmHg), higher C-reactive protein levels (3.1 vs. 2.4 mg/L), and increased glycated hemoglobin (5.4 vs. 5.2%, *p* < 0.001). And individuals in Cluster 2 were less likely to be smokers or drinkers, possibly reflecting functional decline or changes in behavior due to worsening health. These findings suggest that Cluster 2 represents a more clinically vulnerable subgroup, consistent with an advanced frailty phenotype.

### Baseline characteristics of the study population

Baseline characteristics of the study population (*n* = 8,049) stratified by stroke status are presented in Table 1. Among all participants, 768 (9.5%) experienced stroke during the follow-up period. The mean age of participants was 58.4 years (SD: 9.4), with stroke patients being significantly older (61.0 vs. 58.2 years, *p* < 0.001). The majority of participants were female (54.7%) and married (88.8%), with stroke patients having a higher proportion of non-married status (15.0% vs. 10.8%, *p* = 0.001). Regarding lifestyle factors, stroke patients had higher proportions of smoking (42.8% vs. 37.7%, *p* = 0.006) and drinking (41.7% vs. 38.0%, *p* = 0.050). Cardiovascular risk factors, including glycated hemoglobin (5.4 vs. 5.2, *p* < 0.001), systolic blood pressure (136.5 vs. 128.2 mmHg, *p* < 0.001), and C-reactive protein (3.5 vs. 2.5, *p* < 0.001), were significantly higher in the stroke group, while HDL cholesterol was lower (48.2 vs. 51.6, *p* < 0.001). Notably, stroke patients showed consistently higher frailty index scores across all three time points (2011: 6.7 vs. 4.1; 2013: 7.5 vs. 4.5; 2015: 9.4 vs. 5.4; all *p* < 0.001). Cluster analysis revealed that stroke patients were more likely to belong to Cluster 2 (58.9% vs. 24.5%, *p* < 0.001), which represented the higher frailty trajectory group.

**Table 1:** Baseline characteristics of the study population stratified by stroke status.

Characteristics	Level	Overall	Stroke	Non-stroke	*p*
*n*		8,049	7,281	768	
Gender (%)	Female	4,400 (54.7)	4,002 (55.0)	398 (51.8)	0.104
	Male	3,649 (45.3)	3,279 (45.0)	370 (48.2)	
Age (mean [SD])		58.4 (9.4)	58.2 (9.4)	61.0 (8.8)	<0.001
Marital (%)	Married	7,145 (88.8)	6,492 (89.2)	653 (85.0)	0.001
	Non-married	904 (11.2)	789 (10.8)	115 (15.0)	
Education (%)	College or above	209 (2.6)	188 (2.6)	21 (2.7)	0.067
	High school	2,139 (26.6)	1962 (26.9)	177 (23.0)	
	Primary school or below	5,701 (70.8)	5,131 (70.5)	570 (74.2)	
Smoke (%)	No	4,977 (61.8)	4,538 (62.3)	439 (57.2)	0.006
	Yes	3,072 (38.2)	2,743 (37.7)	329 (42.8)	
Drink (%)	No	4,964 (61.7)	4,516 (62.0)	448 (58.3)	0.05
	Yes	3,085 (38.3)	2,765 (38.0)	320 (41.7)	
BMI (mean [SD])		24.0 (29.0)	24.0 (30.5)	24.4 (4.4)	0.715
glycated_hemoglobi (mean [SD])		5.3 (0.8)	5.2 (0.8)	5.4 (0.9)	<0.001
SBP (mean [SD])		129.0 (21.2)	128.2 (20.8)	136.5 (23.5)	<0.001
hdl_cholestero (mean [SD])		51.3 (15.3)	51.6 (15.3)	48.2 (15.0)	<0.001
c_reactive_protein (mean [SD])		2.6 (7.0)	2.5 (6.7)	3.5 (9.0)	<0.001
physical_dysfunction (%)	No	2,514 (31.2)	2,360 (32.4)	154 (20.1)	<0.001
	Yes	5,535 (68.8)	4,921 (67.6)	614 (79.9)	
Frailty index of 2011 (mean [SD])		4.4 (3.6)	4.1 (3.4)	6.7 (4.5)	<0.001
Frailty index of 2013 (mean [SD])		4.8 (3.7)	4.5 (3.5)	7.5 (4.7)	<0.001
Frailty index of 2015 (mean [SD])		5.7 (4.4)	5.4 (4.1)	9.4 (5.5)	<0.001
Cluster (%)	1	5,812 (72.2)	5,496 (75.5)	316 (41.1)	<0.001
	2	2,237 (27.8)	1,785 (24.5)	452 (58.9)	

**Figure 2:**
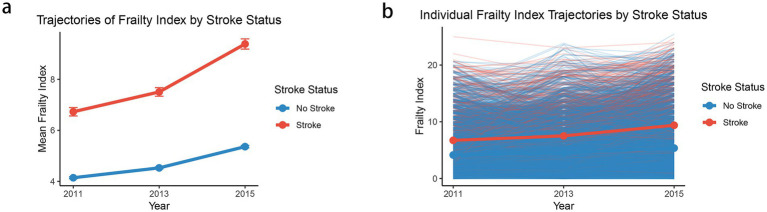
The mean frailty index trajectories **(a)** and individual frailty index trajectories **(b)** by stroke status. Each point in panel **(a)** represents the mean frailty index for the corresponding cluster at a given time point (2011, 2013, and 2015), and the vertical error bars denote the standard error (SE) of the mean.

Figure 2a illustrates the temporal evolution of frailty index scores stratified by stroke status during the follow-up period (2011–2015). The mean trajectory analysis revealed distinct patterns between individuals who experienced stroke (*n* = 768) and those who did not (*n* = 7,281). Participants who subsequently developed stroke demonstrated consistently higher frailty index scores throughout the observation period, with an accelerated increase from baseline (6.7 in 2011) to follow-up (9.4 in 2015). In contrast, the non-stroke group maintained relatively lower frailty levels, showing a more gradual increase from 4.1 in 2011 to 5.4 in 2015. The individual trajectory plot further elucidates the heterogeneity in frailty progression patterns, displaying considerable inter-individual variability within both groups (Figure 2b). While the mean trajectories clearly differentiate the stroke and non-stroke groups, the individual-level data reveal substantial overlap in frailty trajectories, suggesting that additional factors beyond baseline frailty status may influence stroke risk. The divergent trajectories between groups became more pronounced over time, particularly in the latter period of follow-up (2013–2015), indicating that the rate of frailty accumulation may be an important predictor of subsequent stroke events.

### Association of stroke with frailty

In the present study, we found that both frailty trajectory patterns and baseline frailty index were significantly associated with stroke risk. The cluster-based analysis revealed that participants in Cluster 2 (higher frailty trajectory) had a substantially increased risk of stroke compared to those in Cluster 1, with an unadjusted hazard ratio (HR) of 4.10 (95% CI: 3.55–4.74, *p* < 0.001). This association remained robust after adjusting for demographic characteristics and cardiovascular risk factors (adjusted HR: 3.85, 95% CI: 3.26–4.56, *p* < 0.001). Similarly, baseline frailty index showed a significant association with stroke risk, where each unit increase in frailty index was associated with a 15% higher risk of stroke in the unadjusted model (HR: 1.15, 95% CI: 1.14–1.17, *p* < 0.001) and a 14% higher risk in the fully adjusted model (HR: 1.14, 95% CI: 1.12–1.16, *p* < 0.001). Other significant predictors of stroke in the fully adjusted models included smoking (HR: 1.23, 95% CI: 1.05–1.45), systolic blood pressure (HR: 1.011, 95% CI: 1.008–1.014), and HDL cholesterol (HR: 0.988, 95% CI: 0.983–0.992). Notably, each 1 mmHg increase in systolic blood pressure was associated with an approximately 1.1% increase in stroke risk. The model discrimination was good, with concordance indices ranging from 0.67 for the unadjusted cluster model to 0.72 for the fully adjusted model, indicating strong predictive capability. These findings suggest that both the trajectory of frailty and baseline frailty status are important predictors of stroke risk, independent of traditional cardiovascular risk factors (Table 2).

**Table 2:** Associations between frailty measures and incident stroke.

Model	Exposure	HR (95% CI)	*p*-value
Model 1	Cluster	4.10 (3.55–4.74)	<0.001
Model 2	Cluster (Adjusted)^*^	3.85 (3.26–4.56)	<0.001
Model 1	Baseline frailty index	1.15 (1.14–1.17)	<0.001
Model 2	Baseline frailty index (Adjusted)	1.14 (1.12–1.16)	<0.001

* Adjusted for age, marital status, education, smoking, drinking, BMI, glycated hemoglobin, systolic blood pressure, HDL cholesterol, C-reactive protein, and physical dysfunction.

Stratified analyses revealed consistent but varying associations between frailty and stroke risk across different subgroups. The association was particularly pronounced among adults aged <80 years, with both cluster-based (adjusted HR: 3.87, 95% CI: 3.27–4.59) and baseline frailty index approaches (adjusted HR: 1.14, 95% CI: 1.12–1.16) showing strong associations. In contrast, among those aged ≥80 years, the associations were attenuated and did not reach statistical significance, possibly due to survival effects or smaller sample size in this age group. Gender-stratified analyses showed slightly stronger associations in males (cluster-adjusted HR: 4.21, 95% CI: 3.31–5.36; frailty index-adjusted HR: 1.15, 95% CI: 1.13–1.18) compared to females (cluster-adjusted HR: 3.58, 95% CI: 2.84–4.51; frailty index-adjusted HR: 1.13, 95% CI: 1.10–1.16). The relationship between frailty and stroke risk was robust across marital status categories, though slightly stronger among non-married individuals (cluster-adjusted HR: 4.14, 95% CI: 2.64–6.49) compared to married participants (cluster-adjusted HR: 3.81, 95% CI: 3.18–4.57). Notably, the association was strongest among individuals without physical dysfunction (cluster-adjusted HR: 4.90, 95% CI: 3.14–7.65; frailty index-adjusted HR: 1.38, 95% CI: 1.25–1.53), suggesting that frailty might be particularly predictive of stroke risk in apparently healthier individuals. These findings highlight the importance of considering frailty assessment across different population subgroups for stroke risk stratification (Table 3).

**Table 3:** Stratified associations between frailty measures and incident stroke across demographic and health-related subgroups.

Subgroups	Exposure	Unadjusted		Adjusted^*^	
		HR (95% CI)	*p* value	HR (95% CI)	*p* value
Age ≥80					
	Cluster	1.48 (0.42–5.25)	0.544	1.85 (0.33–10.40)	0.487
	Baseline frailty index	1.03 (0.89–1.18)	0.729	1.20 (0.95–1.50)	0.127
Age <80					
	Cluster	4.19 (3.63–4.85)	<0.001	3.87 (3.27–4.59)	<0.001
	Baseline frailty index	1.16 (1.14–1.17)	<0.001	1.14 (1.12–1.16)	<0.001
Male					
	Cluster	4.76 (3.88–5.83)	<0.001	4.21 (3.31–5.36)	<0.001
	Baseline frailty index	1.17 (1.15–1.20)	<0.001	1.15 (1.13–1.18)	<0.001
Female					
	Cluster	3.97 (3.23–4.87)	<0.001	3.58 (2.84–4.51)	<0.001
	Baseline frailty index	1.14 (1.12–1.17)	<0.001	1.13 (1.10–1.16)	<0.001
Married					
	Cluster	4.11 (3.52–4.79)	<0.001	3.81 (3.18–4.57)	<0.001
	Baseline frailty index	1.16 (1.14–1.18)	<0.001	1.14 (1.12–1.17)	<0.001
Non-married					
	Cluster	3.78 (2.54–5.65)	<0.001	4.14 (2.64–6.49)	<0.001
	Baseline frailty index	1.12 (1.08–1.16)	<0.001	1.12 (1.08–1.17)	<0.001
Non-smoker					
	Cluster	4.16 (3.43–5.05)	<0.001	3.94 (3.15–4.92)	<0.001
	Baseline frailty index	1.16 (1.14–1.18)	<0.001	1.15 (1.12–1.17)	<0.001
Smoker					
	Cluster	4.29 (3.45–5.32)	<0.001	3.85 (2.98–4.96)	<0.001
	Baseline frailty index	1.15 (1.13–1.18)	<0.001	1.13 (1.11–1.17)	<0.001
Non-drinker					
	Cluster	3.85 (3.19–4.65)	<0.001	3.44 (2.77–4.28)	<0.001
	Baseline frailty index	1.15 (1.13–1.18)	<0.001	1.14 (1.12–1.17)	<0.001
Drinker					
	Cluster	4.69 (3.76–5.86)	<0.001	4.54 (3.49–5.91)	<0.001
	Baseline frailty index	1.15 (1.13–1.18)	<0.001	1.14 (1.11–1.17)	<0.001
Physical dysfunction-Yes					
	Cluster	3.91 (3.30–4.64)	<0.001	3.77 (3.16–4.51)	<0.001
	Baseline frailty index	1.15 (1.13–1.17)	<0.001	1.14 (1.12–1.16)	<0.001
Physical dysfunction-No					
	Cluster	6.43 (4.25–9.75)	<0.001	4.90 (3.14–7.65)	<0.001
	Baseline frailty index	1.50 (1.36–1.65)	<0.001	1.38 (1.25–1.53)	<0.001

* Adjusted for age, marital status, education, smoking, drinking, BMI, glycated hemoglobin, systolic blood pressure, HDL cholesterol, C-reactive protein, and physical dysfunction (except for the stratification variable).

## Discussion

In this large-scale longitudinal study of 8,049 Chinese older adults, we found robust associations between frailty and stroke risk using both trajectory-based and baseline assessments of frailty. Our findings demonstrate that individuals with higher frailty trajectories (Cluster 2) had nearly four times the risk of stroke compared to those with lower trajectories (adjusted HR = 3.85, 95% CI: 3.26–4.56), even after adjusting for traditional cardiovascular risk factors. This substantial increase highlights the potential utility of frailty trajectory monitoring as a clinically meaningful screening tool for identifying high-risk individuals. Similarly, the baseline frailty index showed a significant association with stroke risk, with each unit increase corresponding to a 14% higher risk (adjusted HR = 1.14, 95% CI: 1.12–1.16), indicating that even static frailty measures hold predictive value. These associations remained consistent across various demographic and health-related subgroups.

Our findings extend the current understanding of frailty-stroke relationships in several important ways. First, the use of trajectory-based analysis provides novel insights into how patterns of frailty accumulation, rather than just point estimates, influence stroke risk. The stronger association observed with the cluster-based approach (HR = 3.85) compared to the baseline frailty index (HR = 1.14) underscores the importance of incorporating temporal dynamics into frailty assessment. In practical terms, a nearly four-fold elevated stroke risk suggests that tracking frailty progression over time may offer superior predictive power, and could be integrated into early intervention strategies aimed at preventing stroke in vulnerable aging populations. This aligns with the conceptual understanding of frailty as a dynamic process rather than a static state. The link between frailty and stroke may be explained by several biological and behavioral mechanisms. Chronic low-grade inflammation, commonly observed in frail individuals, has been implicated in both vascular aging and cerebrovascular disease. In addition, frailty-related conditions such as sarcopenia, immobility, and poor nutritional status can contribute to reduced cerebral perfusion, increased oxidative stress, and vascular dysfunction, thereby elevating stroke risk. Cognitive decline and impaired self-care associated with frailty may also delay the management of traditional risk factors such as hypertension and diabetes. These pathways suggest that frailty is not merely a marker of vulnerability but may actively contribute to stroke pathogenesis.

Importantly, it should be noted that both frailty assessment methods—cluster-based frailty trajectory and baseline frailty index—were evaluated using identical Cox model structures and the same set of covariates. The observed difference in predictive strength (HR = 3.85 vs. 1.14) therefore likely stems from the different nature of the frailty measures themselves. The trajectory-based cluster approach encapsulates longitudinal changes in frailty status, providing a more dynamic representation of health decline over time, whereas the baseline frailty index reflects only a single time-point assessment. These differences underscore the added value of monitoring frailty progression longitudinally in enhancing risk stratification for stroke.

The subgroup analyses revealed important variations in the frailty-stroke relationship. The stronger associations observed in younger elderly (<80 years) suggest that frailty might be particularly important for early stroke risk prediction. The attenuated association in the ≥80 age group might reflect survival effects or the competing risks of other age-related conditions. The gender differences in association strength, with stronger effects in males, could reflect different pathophysiological mechanisms or varying impacts of frailty components between sexes (21, 22). Particularly noteworthy was the strongest association found among individuals without physical dysfunction, suggesting that frailty assessment might be especially valuable for risk stratification in apparently healthy older adults. This finding challenges the common clinical practice of focusing frailty assessments primarily on obviously impaired individuals and suggests potential benefits of broader screening approaches. The observed associations between frailty and stroke remained robust after adjusting for traditional cardiovascular risk factors, indicating that frailty captures additional risk information beyond conventional predictors. This supports the notion that frailty represents a distinct pathophysiological pathway to stroke, possibly through mechanisms such as chronic inflammation, endothelial dysfunction, or alterations in cellular aging processes (23, 24). The significant associations with traditional risk factors in our models (smoking, blood pressure, and HDL cholesterol) also validate the overall quality of our risk assessment approach.

Although the longitudinal design of this study helps to establish a temporal sequence between frailty assessment and stroke onset, the possibility of reverse causality cannot be fully excluded. Frailty, especially when assessed using multidimensional indices, may partly reflect the presence of subclinical cerebrovascular pathology, such as silent infarcts or microvascular changes, that precede overt clinical stroke. These underlying pathologies could simultaneously contribute to frailty progression and increase future stroke risk, potentially biasing the observed association. Future studies incorporating neuroimaging or biomarker data would be valuable in disentangling this bidirectional relationship.

Our results have important clinical and public health implications. The strong predictive value of frailty trajectories suggests that regular monitoring of frailty status could enhance stroke risk assessment in older adults. The varying associations across subgroups indicate the need for targeted screening and prevention strategies. For instance, the stronger associations in males and non-married individuals might warrant more intensive monitoring in these populations. Importantly, these findings also highlight the potential for early intervention to mitigate frailty progression and reduce stroke risk. Interventions such as resistance training, nutritional supplementation (e.g., protein and vitamin D), and cognitive engagement (e.g., memory exercises, social participation programs) have shown promise in previous studies and may be effective in slowing the accumulation of frailty. Tailored multimodal interventions that combine physical, nutritional, and cognitive components could be particularly beneficial for high-risk individuals. Future research should explore the feasibility, timing, and cost-effectiveness of such strategies in the Chinese aging population.

These findings have some implications for real-world practice, particularly in primary care and community health settings where early identification of at-risk individuals is critical. Routine frailty screening—using either brief frailty indices or trajectory-informed risk models—could be integrated into annual health check-ups for older adults to identify those at elevated stroke risk. The trajectory-based approach, in particular, highlights the importance of repeated frailty assessments over time, rather than relying solely on single-time-point evaluations. In primary care, implementing longitudinal frailty monitoring could be achieved through electronic health record (EHR)-based alerts or automated risk flags triggered by worsening frailty scores. Such strategies could enable proactive intervention—e.g., tailored exercise programs, blood pressure control, or behavioral support—before overt stroke symptoms emerge. Our findings thus support the integration of frailty surveillance into stroke prevention protocols as part of holistic aging care.

While these findings are encouraging, several limitations warrant consideration. First, while CHARLS provides high-quality longitudinal data, the relatively short follow-up period (5 years) may not capture the full temporal relationship between frailty and stroke risk. Second, stroke cases were ascertained via self-reported physician diagnoses, which has demonstrated reasonable accuracy in prior large-scale surveys, this method remains subject to potential misclassification or underreporting. In particular, stroke events that were not clinically diagnosed or inadequately recalled may have been missed, potentially biasing the association estimates toward the null. Future research leveraging linkage with medical records or insurance claims could help validate and refine self-reported stroke outcomes. Third, while we adjusted for multiple confounders, residual confounding from unmeasured factors cannot be ruled out. Fourth, the study’s findings might not be fully generalizable to non-Chinese populations due to potential differences in healthcare systems, lifestyle factors, and genetic backgrounds. Differences in genetic backgrounds, cultural norms, dietary patterns, physical activity levels, and access to healthcare services may influence both frailty progression and stroke risk. For instance, the prevalence of multigenerational households and traditional caregiving roles in Chinese culture may affect frailty dynamics differently compared to Western contexts. Additionally, variations in primary care infrastructure and preventive health policies could modify the observed associations. Therefore, caution is warranted when extrapolating these findings to populations with different sociodemographic or healthcare system characteristics. Future research in diverse ethnic and geographic settings is needed to validate and expand upon these results. However, given that CHARLS is a nationally representative dataset from one of the world’s most rapidly aging societies, the observed associations may still offer valuable insights for other countries facing similar demographic and epidemiological transitions. Future research should focus on several key areas. Longer-term follow-up studies are needed to better understand the temporal dynamics of the frailty-stroke relationship. Investigation of potential mechanistic pathways linking frailty to stroke risk could inform targeted interventions. Additionally, studies examining whether frailty-based risk stratification can improve clinical outcomes through early intervention would be valuable for translating these findings into practice.

## Conclusion

Our findings demonstrate that both trajectory-based and baseline assessments of frailty are strong predictors of stroke risk in Chinese older adults, independent of traditional risk factors. The stronger associations observed with trajectory-based assessment suggest the value of longitudinal frailty monitoring in stroke risk prediction. These results support the incorporation of frailty assessment into stroke risk stratification strategies and highlight the importance of considering frailty patterns in the prevention and management of stroke in aging populations.

Taken together, our findings underscore the clinical relevance of incorporating dynamic frailty assessments—such as longitudinal trajectory monitoring—into stroke risk prediction frameworks. By identifying individuals at elevated risk earlier and more accurately, such approaches may support more personalized, proactive prevention strategies in aging populations. Future research should aim to validate these findings in diverse populations and explore implementation pathways in primary care settings.

## Data Availability

The original contributions presented in the study are included in the article/Supplementary material, further inquiries can be directed to the corresponding authors.
